# New national osteoporosis guidance—implications for geriatricians

**DOI:** 10.1093/ageing/afac044

**Published:** 2022-04-11

**Authors:** Celia L Gregson, Juliet E Compston

**Affiliations:** Musculoskeletal Research Unit, Bristol Medical School, University of Bristol, Bristol, BS10 5NB, UK; Older Person’s Unit, Royal United Hospital NHS Foundation Trust, Bath, BA1 3NG, UK; Cambridge Biomedical Campus, University of Cambridge, Cambridge, CB2 0AH, UK

**Keywords:** osteoporosis, fragility fracture, fracture risk, NOGG, FRAX, older people, clinical guidelines

## Abstract

Fragility fractures are painful, debilitating, often life-changing and accounted for an estimated 2.4% of pre-pandemic health care spending in the UK. Those who are older, frail and multimorbid have the highest fracture risk and therefore the most to gain from anti-osteoporosis treatments to reduce this risk. Currently, an unacceptable treatment gap exists between those eligible for and those who receive treatment. This commentary discusses the major changes to the new, National Institute for Health and Care Excellence accredited, UK National Osteoporosis Guideline Group (NOGG) guidance (published March 2022) most relevant to the management of older people’s bone health. Changes include intervention thresholds; using fracture probabilities from FRAX; for patients too frail to undergo DXA; greater emphasis on vertebral fracture detection and the use of intravenous zoledronate as a first-line anti-osteoporosis therapy; the new concept of ‘very high fracture risk’ which should prompt consideration of use of parenteral anti-osteoporosis therapy; new guidance regarding anabolic treatment options; concerns regarding denosumab cessation; and the urgent need to get patients with a fragility fracture onto treatment to reduce re-fracture risk with follow-up to check tolerance and ensure adherence.

## Key Points

- All older people we see in clinic and on our wards should have their fracture risk assessed as part of comprehensive geriatric assessment.
- All radiographic imaging that visualises the spine should be routinely reviewed to detect incidental vertebral fractures.
- Offer older people with a fragility fracture prompt anti-osteoporosis treatment because of their high re-fracture risk.
- For those too frail to have a DXA, new post-FRAX intervention thresholds indicate whom to treat.
- ‘Very high fracture risk’ is indicated by ‘red flags’, consider using parenteral anti-osteoporosis treatments here.
- When initiating oral bisphosphonates in older patients, counsel them for 10 years of treatment.
- Denosumab cessation needs careful planning due to the increased risk of vertebral fractures.

## Introduction

In March 2022, the UK National Osteoporosis Guideline Group (NOGG) published the new UK guideline for the assessment and management of osteoporosis and the prevention of fragility fractures in postmenopausal women and men aged ≥50 years [1, 2]. This evidence-based guideline, accredited by the National Institute for Health and Care Excellence (NICE), represents a major update to the 2017 NOGG guideline and is highly relevant to geriatricians and other health professionals managing older people’s health.

Currently, approximately 549,000 new fragility fractures occur each year in the UK (a third in men); with population ageing, a 19.6% increase is expected by 2030 [3]. Pre-pandemic, fragility fractures were estimated to cost the NHS £5.4 billion a year, accounting for 2.4% of health care spending [4]. Of concern is the current anti-osteoporosis treatment gap; analysis of UK GP prescribing data shows that 1 year after a fragility fracture, only a third of eligible patients (mean age: 79 years) are on anti-osteoporosis treatment, and this gap is worse for men [5]. More than half of patients with hip fracture will have had a prior fragility fracture, yet at hip fracture presentation, only 12% are on any bone medication [6, 7]. This article discusses the major changes to the new NOGG guidance most relevant to the management of older people’s bone health.

## Fracture risk assessment in frail older people

As most people who sustain a fragility fracture have a femoral neck BMD T-Score >−2.5 [8], consideration of the many clinical risk factors (CRFs) besides BMD, for fragility fracture risk, is crucial. Any single CRF for fracture should prompt assessment of fracture risk, e.g*.* frailty, sarcopenia, a fall, a stroke, diabetes, dementia, depression, Parkinson’s disease and many more [1, 2]. Given age-associated morbidity levels, practically this means that all inpatients and outpatients seen by a geriatrician should have their bone health considered as routine; fracture risk assessment should form part of routine comprehensive geriatric assessment (CGA) [9].

NOGG continues to recommend the use of FRAX rather than Qfracture to assess fracture risk. While Qfracture can indicate fracture risk over shorter timeframes than 10 years, and includes a greater number of CRFs than FRAX, it is lengthier to complete, cannot incorporate BMD measurement and tends to overestimate major osteoporotic fracture (MOF) risk compared with FRAX [10]. NOGG now recommends a number of new arithmetic adjustments to FRAX-derived fracture risk probabilities to take account of additional CRFs for fracture not included in FRAX, e.g*.* type II diabetes and, importantly, falls history [11]. NOGG recommends that for patients with two or more falls in the last year, MOF and hip fracture probabilities should be increased by 30% above those generated by FRAX [1, 2].

## Intervention thresholds recommended by NOGG

Certainly, treatment should be considered for any older person who sustains a fragility fracture, and this remains unchanged in the updated NOGG guidance. After using FRAX, NOGG now defines four categories of fracture risk based on FRAX outputs: low risk (green), intermediate risk (amber), high risk (red) and very high risk (dark red), with DXA recommended in all patients with intermediate or higher risk [1, 2].

Hitherto, a common dilemma for geriatricians has arisen when fracture risk is intermediate, but BMD measurement has been impractical due to frailty. Now a strong recommendation is made to offer anti-osteoporosis treatment if there is a history of fragility fracture and/or if fracture risk exceeds the intervention thresholds set at 20.3% for MOF and at 5.4% for hip fracture; corresponding lines are now clearly shown on post-FRAX NOGG graphs [2].

## Very high fracture risk

The concept of ‘very high fracture risk’ is new and is indicated by the presence of one or more important CRFs, or ‘red flags’, such as a recent (within 2 years) vertebral fracture, or ≥ 2 vertebral fractures (whenever they happened), a BMD T-Score ≤−3.5, treatment with high dose glucocorticoids (≥7.5 mg/day of prednisolone or equivalent over 3 months), the presence of multiple CRFs particularly with a recent fragility fracture which indicates high imminent risk of re-fracture, or other indicators of very high fracture risk, such as by FRAX assessment. As age is such a strong predictor of fracture risk, it is estimated that 32.5% of people aged ≥70 years will be at very high fracture risk based on FRAX estimates [1, 2]. A conditional recommendation is made: those with very high risk should be considered for referral to an osteoporosis specialist in secondary care, for assessment and consideration of parenteral treatment. Without consideration, this could translate to a very high number of secondary care referrals. The guideline explains a conditional recommendation applies ‘where the clinician examines the evidence within the wider health and social context and discusses the choices with the patient, taking into account the patient’s values and preferences, or where documentation of the discussion of the pros and cons of an intervention is the indicator of quality, rather than the course of action itself’ [1, 2]. This patient-centred holistic approach to decision-making will be very familiar to geriatricians. It is important when deciding on a treatment pathway to consider the patient’s wishes, their ability to attend a hospital, the fact that delays in treatment initiation (while waiting for a hospital appointment) increase fracture risk as well as the potential benefit of the treatment options.

Parenteral therapies, to reduce very high fracture risk, include antiresorptive (intravenous zoledronate and subcutaneous denosumab) and anabolic agents (subcutaneous teriparatide and biosimilars, and potentially romosozumab). The emphasis on earlier parenteral treatment is new, and reflects (i) well-recognised adherence challenges with oral bisphosphonates, (ii) the unacceptable treatment gap currently evident in the UK (see above), (iii) high clinical and cost effectiveness of zoledronate, (iv) a strong evidence-base to support use of denosumab out to 10 years and, importantly, (v) the increasing range of anabolic anti-osteoporosis treatments which are first-line options in some very high risk individuals, especially those with multiple vertebral fractures.

The recommendation to consider referral when high doses of glucocorticoids are planned reflects the fact that bone is lost rapidly when glucocorticoids are started, hence fracture risk increases sharply. NOGG emphasises that if any delay is anticipated in the referral pathway, an oral bisphosphonate should be started in the meantime [1, 2].

## Treatment approaches

Oral alendronate remains a highly clinically effective and cost-effective first-line anti-osteoporosis treatment, which is suitable for the majority of older people with high fracture risk, and, after consideration, for many with very high fracture risk unsuitable for parenteral intervention. Notably, because older people have such high absolute fracture risks, the number needed to treat (NNT) is relatively few; in a `real-world' setting in Sweden, within a population aged ≥80 years recruited from hospitals and care homes, the NNT with oral alendronate to prevent a hip fracture was 26, and any fracture 20, compared against a number needed to harm, by causing mild upper gastrointestinal symptoms, of 91 [12].

Treatment duration and monitoring guidance for people treated with oral bisphosphonates have been tightened. Anyone, either aged 70 years, or with ≥2 vertebral fractures, or is taking glucocorticoids, should be counselled from the outset for 10 years of treatment (only those who are younger and at lower risk should be considered for a treatment pause after 5 years). Tolerance to oral bisphosphonates must be checked after 12–16 weeks, adherence must be checked after 1 year and fracture risk must be reassessed after 5 years (sooner if re-fracture or new CRFs develop) [1, 2]. This change in emphasis responds to the increased fragility fracture risk seen when anti-resorptive therapy is routinely stopped after 5 years; a decision in higher-risk patients that often fails to balance the far greater risk of typical fragility fracture versus very rare side-effects [13].

**Figure 1:**
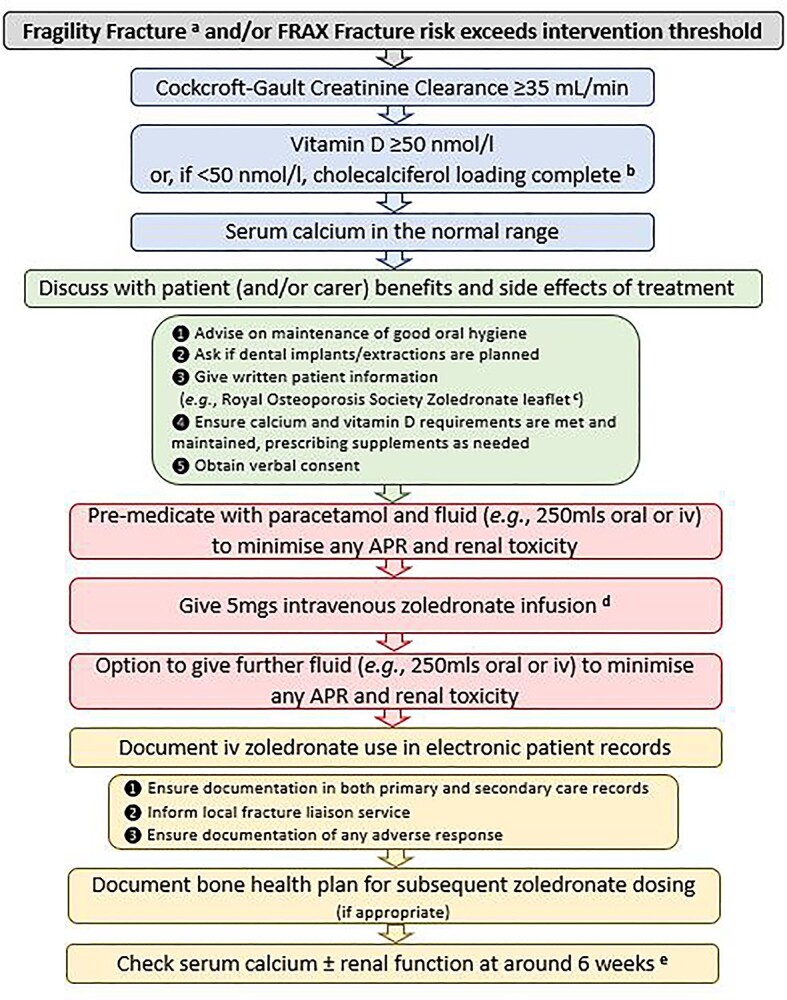
Flow diagram showing process of giving intravenous zoledronate to older people. APR: Acute Phase Reaction, iv: intravenous. ^a^: If an orthopaedic operation has been performed, there is minimal post hoc evidence to support delaying zoledronate administration to ≥2 weeks [22], so it is important to balance the benefit of ensuring prompt treatment of imminent fracture risk against waiting the SmPC-advised 2 weeks. ^b^: an example of a rapid cholecalciferol loading schedule: provided there is no hypercalcaemia, if serum 25OHD < 25 nmol/l, prescribe 300,000 iu of cholecalciferol, e.g*.* as 50,000 iu alternate daily; if 25–50 nmol/l, prescribe 150,000 iu of cholecalciferol, e.g*.* as 25,000 iu alternate daily; if cholecalciferol replacement is documented as given, there is no need to recheck the serum 25OHD level prior to administration if zoledronate. ^c^: https://theros.org.uk/information-and-support/osteoporosis/treatment/zoledronic-acid/). ^d^: Some advocate giving slowly over one hour to reduce risk of APR; evidence is needed to support this. ^e^: To check no hyperparathyroidism unmasked by vitamin D replacement. Check renal function if there is pre-existing renal impairment.

Intravenous zoledronate is now considered as a first-line anti-osteoporosis treatment, particularly post-hip fracture (Figure 1 provides an example of the process of giving zoledronate to older people). Approximately, 25% of hip fracture patients will go on to fracture again over 5 years [14], with half of these re-fractures occurring within 18 months [15], so prompt treatment is essential. A single infusion of zoledronate leads to a 23% clinical fracture reduction (HR: 0.77 [0.57–1.03]; *P* = 0.080) by 6 months and 25% (HR: 0.75 [0.61–0.92]; *P* = 0.005) by 12 months [16], hence clinical benefit can be realised even in those with a challenged life expectancy, potentially preventing a painful inpatient death. Frailty can restrict the ability to attend secondary care services for annual (or 18-monthly [17]) infusions, making a single inpatient zoledronate infusion all that may be logistically feasible (following rapid vitamin D loading as needed). Encouragingly, those patients receiving only one zoledronate infusion in the HORIZON studies saw similar relative fracture risk reductions to those who received the three planned annual doses, which included an impressive 68% reduction in vertebral fractures, suggesting that much of the antifracture effect of zoledronate is conveyed by the first dose [18]. However, in the UK, currently only 9% of hip fracture patients receive zoledronate before discharge, although this varies substantially between hospitals, ranging from 0 to 63% [7]. It is very much hoped that the new NOGG guidance will lead to more equitable use of this highly effective treatment. Promisingly, successful nurse-lead community-delivered zoledronate programmes have been reported [19].

Recent development of generic teriparatide and biosimilars, plus romosozumab approval, has rapidly expanded anabolic treatment options for those with severe osteoporosis at the highest fracture risk. Given the strong trial evidence supporting the clinical effectiveness of these anabolic agents, particularly in postmenopausal women with vertebral fractures, NOGG makes a conditional recommendation for anabolic treatment to be considered first-line in postmenopausal women at very high fracture risk, especially those with vertebral fractures (in men, only teriparatide is recommended as romosozumab lacks an evidence base), thereafter consolidated by an anti-resorptive [1, 2]. However, the need for daily subcutaneous teriparatide injections limits uptake, and while romosozumab only requires monthly subcutaneous injection, its contraindication in those with a history of myocardial infarction or stroke limits use in older populations. The high costs of generic teriparatide and the biosimilars, as well as the projected £5,133 per year cost for romosozumab, are a challenge. Disappointingly, NICE’s initial (December 2021) decision regarding romosozumab was negative.

## Denosumab initiation and cessation

Patients discontinuing denosumab have an increased risk of multiple vertebral fractures, on average, 3-6 months since the last denosumab injection is due, which is attributable to a rapid increase in bone turnover and accelerated bone loss. Hence NOGG strongly emphasises that denosumab doses must not delayed, nor should denosumab be stopped without considering an alternative therapy; intravenous zoledronate is recommended 6 months after the last denosumab injection with serum CTX monitoring to guide timing of further treatment, or a further infusion of zoledronate 6 months later [1, 2]. In older people with impaired renal function, zoledronate may be contraindicated and there is little evidence to support an alternative approach. Therefore, before starting denosumab, it is crucial that a long-term osteoporosis management plan is agreed with the patient and GP. Trial data show denosumab continues to increase BMD and reduces fracture risk up to 10 years if not stopped [20], However, even when initiating denosumab in patients thought to be in the last decade of life, a long-term osteoporosis management plan is needed in case unplanned cessation becomes necessary.

## Vertebral fractures

Much greater emphasis is placed on the clinical importance of vertebral fractures in this new guideline, given they (i) are common and often overlooked on routine radiological imaging (two-thirds fail to come to clinical attention), (ii) cause acute and chronic pain, functional impairment and reduce quality of life and (iii) predict high imminent re-fracture risk. Identification of vertebral fractures offers an important opportunity to intervene to prevent further (e.g*.* hip) fractures.

NOGG recommends diagnostic imaging services should routinely evaluate the vertebrae in all imaging of all older people in which the spine is visualised, reporting vertebral fractures using standardised language [1, 2]. Until this is an established practice, routine visualisation of vertebrae on thoracic and abdominal imaging would ideally form part of fracture risk assessment within CGA.

Acute onset back pain in older people necessitates imaging. NOGG presents the evidence-based supporting interventions for acute vertebral fracture management, including strong evidence supporting physiotherapist-supervised exercise [1, 2]. More broadly, NOGG supports exercise interventions for bone and muscle health, including the Royal Osteoporosis Society ‘Strong, Steady and Straight’ Expert Consensus Statement [21].

## Further NOGG resources

The guidance includes recommendations regarding fracture liaison services, training of clinicians and allied health professionals, service commissioning and criteria for audit and quality improvement and is complemented by ‘frequently asked questions’ (FAQs) and responses on the NOGG website [2], where further FAQs can also be proposed.

## Conclusions

Geriatricians manage a patient population with the highest fragility fracture risk and in whom such fractures are often devastating. Currently, there is a large treatment gap that deprives many individuals at high risk of fracture from receiving effective anti-osteoporosis treatment. Improved case finding, by routine fracture risk assessment, and prompt treatment delivery as part of routine CGA are essential. Fall and fractures risk assessments are both integral components of management; assessment of one necessitates the other.

Fortunately, we have an expanding range of anti-osteoporosis treatments. The recommendation to use zoledronate as a first-line option enables prompt inpatient and/or day-case treatment, alleviating polypharmacy. Our services will need to expand. Innovative service delivery models to routinely deliver parenteral treatments within communities, primary care or at home are an exciting and much needed development.
